# Teenage Blood Donors: Are We Asking Too Little and Taking Too Much?

**DOI:** 10.1542/peds.2016-2955

**Published:** 2017-03-03

**Authors:** Evan M. Bloch, Alan E. Mast, Cassandra D. Josephson, Harvey G. Klein, Anne F. Eder

**Affiliations:** a Department of Pathology, Johns Hopkins University, School of Medicine, Baltimore, Maryland; b BloodCenter of Wisconsin, Milwaukee, Wisconsin; c Pathology and Pediatrics Center for Transfusion and Cellular Therapies, Aflac Cancer Center and Blood Disorders Service, School of Medicine, Emory University, Atlanta, Georgia; d National Institutes of Health Clinical Center, Bethesda, Maryland

Young blood donors contribute substantially to the US blood supply, yet these are the donors at greatest risk of immediate reactions and other adverse health effects related to their blood donations. Sixteen and 17-year-old adolescents constitute only 2.8% of the US population, but they contribute an estimated 10% of the blood supply, with more than 1 million donations per year.^1^ Mass recruitment and blood collection are scheduled at high schools despite the consistent demonstration that teenage blood donors are at significantly increased risk of phlebotomy-related reactions and injuries after blood donation as compared with adults.^2^ Approximately one-third of all donor reactions and more than half of all syncope-related injuries occur in adolescents and young adults.^2^ In addition to the immediate hazards of the donation process, iron deficiency has become well recognized as a complication of frequent blood donation, and high school–age students are particularly vulnerable. How did a strategy of deliberate and disproportionate blood collection from young donors come to be, and should this be abandoned or at least modified?

Blood collection is stringently regulated in the United States. Blood centers must conform to federal regulations and most adhere to additional voluntary accreditation standards. Consequently, numerous precautions have been instituted both to protect the donors' health and to ensure the safety of the blood supply. Young age is the strongest independent predictor of adverse reactions, yet, specific eligibility criteria have not been defined in federal regulations or accreditation standards beyond those that apply to all donors, such as weight, blood pressure, heart rate, and temperature. Federal regulations are silent about the minimal acceptable age for donation. The AABB (formerly the American Association of Blood Banks), the major accrediting agency for blood banks, has set the minimum donation age at 16 years or as defined in applicable state law. Left to individual state legislatures, different policies have emerged. In 2006, only 6 states permitted 16-year-olds to donate blood, but many others quickly followed suit, spurred at least in part by blood center advocacy in response to escalating blood use at the time. Today, the laws in 37 states and the District of Columbia allow 16-year-olds to donate blood; 5 states do not require parental consent. Almost all states allow 17-year-olds to donate. Only 5 states require either written parental consent or parental notification. Consequently, many parents are not informed and may be unaware of their child's decision to donate blood and its implications.

Although most blood donations are uneventful, >10% of young donors will experience minor symptoms, typically limited to dizziness or localized bruising at the phlebotomy site.^2^ Young donors are 14-fold more likely than adults to experience serious injuries after blood donation, even after controlling for age and other factors that contribute to higher reaction rates.^2^ Complications include syncope-related falls with head injuries, facial lacerations, dental injuries, and fractures. Although the rates of syncope (4 per 1000) and related injuries (0.6 per 1000) are low, an estimated 5000 high school students will lose consciousness and 600 will incur injuries after blood donation each year. Furthermore, iron depletion remains an underappreciated risk of blood donation, particularly for juveniles. A single whole-blood donation reduces stores by ∼230 mg of elemental iron, requiring >6 months for recovery in the absence of supplementation.^3^ Iron status is not part of the screening procedure. Instead, eligibility to donate is based on a hemoglobin value, which is preserved until iron stores become totally depleted.^4^ Furthermore, borderline or depleted iron stores have been shown to impair cognitive function in children and young women even in the absence of anemia.^5^ The immediate or potential long-term effects in adolescents have not been assessed.

Nonetheless, high school students currently comprise an integral part of the blood donor pool in the United States. The relative ease of collection via high school drives, a low prevalence of transfusion-transmissible infections, and a high degree of prosocial motivation (“altruism”) have made them a desirable target group for blood centers and encourage their continued recruitment. Early age of donation is also thought to predict donor retention by instilling long-term loyalty and a commitment to blood donation. High-yield, low-cost high school blood drives present persuasive economic arguments to continue or expand these programs. Furthermore, the steady decline in blood use and challenging reimbursement mechanisms have led to a national concern about the financial stability of US blood collectors.^6^ Loss of high school blood drives would additionally raise operational costs and place additional stress on blood centers. Strategies to mitigate the risks to juvenile blood donors should also take into account the effect on the nation's blood resource.

There are at least 3 options, which are not mutually exclusive, to address the documented increased risk in young blood donors: (1) raise the minimum donation age; (2) modify informed consent practices to involve parents; and (3) improve safety within the extant donation practice. The most controversial stance is to shift policy and raise the age of eligibility to at least 17 years, and possibly 18 years. The operational and economic impact on blood collectors will likely fuel strong resistance. Rapid implementation could stress the ability of blood centers to meet immediate patient needs and impair surge capacity in blood inventories in response to disasters. However, a continuing shortfall in the provision of blood is unlikely: current survey data suggest that 16-year-olds are not critical to maintain an adequate supply. Transfusion needs were adequately met before the lowering of the donation age at a time when the available blood supply was almost 20% higher than today (16.0 million units in 2006^7^ versus 13.4 million units in 2013^8^). However, the cost of collections would likely increase and some centers would experience financial stress.

The second consideration and a recognized measure to increase awareness, albeit one that has not been implemented widely, is providing educational materials to parents about blood donation and its possible risks. Parents should be involved in decisions that could affect their children's health. However, education alone is insufficient in the case of minors. A parental informed consent process specific to the risks of donation for adolescents should be developed and evaluated. Its use should be encouraged for all adolescents donating at high school drives.

The third approach is to identify measures that improve safety for young donors. The need for research into physiologic and psychological factors within the adolescent blood donor population that might reduce the risk of acute reactions and minimize the possibility of iron depletion has been recognized. Several such measures have already proved effective. The adoption of stringent criteria specific to high school drives (ie, donor height and weight), which select teenagers who are more physically developed than their peers, has reduced the reaction rate by ∼20%, although the rate of injuries remains unaffected.^9^ Strategies are also available to reduce depletion of iron stores. Adolescents are still in a phase of active growth and development, rendering them particularly susceptible to the effects of low iron. Approaches to mitigate iron deficiency in blood donors have been extensively reviewed and include extending the interdonation interval, screening using ferritin testing to assess iron stores, and provision of iron supplementation.^10^ The last of these would require parental permission in the case of minors. Some blood centers are reluctant to introduce iron supplementation because of perceived liability if injury or overdose were to occur, potential masking of anemia from causes unrelated to phlebotomy, and donor intolerance of oral iron with reluctance to return for future donations. Other centers argue that providing iron supplementation converts volunteer donors into patients and ends up treating a condition that is better avoided. These concerns are legitimate, but also open to study.

Teenage blood donors are currently assuming unequal and unnecessary risks under the umbrella of altruism. Minors merit additional safeguards when recruited for blood donation. Donor age is critical to donor selection and safety, yet suitability has been left to determination by state legislatures. Uniform standards should be considered either by federal regulators or by professional societies, such as the AABB and the American Academy of Pediatrics. A standardized approach to safety and a rigorous informed consent process are moral imperatives. The current practices place many adolescent donors at disproportionate risk. Inaction is not an acceptable alternative.
